# Persistent monocyte activation and remodeling in people with HIV after 36 months of switching from a 3-drug regimen to B/F/TAF or DTG/3TC: a sub-study of the DEBATE trial

**DOI:** 10.3389/fimmu.2026.1957183

**Published:** 2026-10-14

**Authors:** Elena Santacroce, Alin Liviu Ciobanu, Lara Gibellini, Domenico Lo Tartaro, Sara De Biasi, William Gennari, Enrica Roncaglia, Barbara Beghetto, Giulia Nardini, Cinzia Puzzolante, Jovana Milic, Andrea Cossarizza, Cristina Mussini

**Affiliations:** 1 Department of Medical and Surgical Sciences for Children and Adults, University of Modena and Reggio Emilia, Modena, Italy; 2 Virology Unit, Azienda Ospedaliero-Universitaria Policlinico, Modena, Italy; 3 Department of Surgery, Medicine, Dentistry and Morphological Sciences, University of Modena and Reggio Emilia, Modena, Italy

**Keywords:** ART, flow cytometry, HIV, immune senescence, monocytes, viral reservoir

## Abstract

**Introduction:**

The DEBATE study is a randomised trial comparing standard three-drug antiretroviral therapy (ART) with either a two-drug regimen (DTG/3TC) or an alternative three-drug regimen (B/F/TAF) in people with HIV who are virally suppressed. While these regimens are effective in controlling the virus, their impact on the innate immune system is less well understood. Monocytes play a pivotal role in inflammation by bridging innate and adaptive immunity and maintaining the viral reservoir. Persistent monocyte activation in individuals treated with ART, as indicated by altered subsets, proinflammatory cytokines and soluble markers, has been associated with chronic inflammation and non-AIDS comorbidities. This highlights the clinical relevance of monocyte activation even when viral suppression is achieved.

**Methods:**

Eleven virally suppressed people with HIV (PWH) randomized to switch to dolutegravir/lamivudine (DTG/3TC, n=6) or to bictegravir/emtricitabine/tenofovir alafenamide (B/F/TAF, n=5) were studied. Peripheral blood mononuclear cells (PBMC) collected at baseline and 36 months after the switch were analyzed using a 17-parameter flow cytometry panel to characterize monocyte phenotype.

**Results:**

Monocyte remodeling in chronically infected, ART-treated PWH involved chemokine receptors such as CX3CR1, CCR5 and CXCR4, which play important roles in cell migration, inflammation and viral reservoir maintenance. Moreover, the upregulation of TAM receptors (MerTK and Tyro3) and CD38 might enhance inflammatory effects in people switching to another three-drug regimen (3DR, B/F/TAF) rather than in those switching to a two-drug regimen (2DR, DTG/3TC). Nonetheless, in DTG/3TC-treated people, inflammation and viral reservoir maintenance might be promoted by the pSTAT3 pathway.

**Discussion:**

Persistent monocyte activation and immune senescence remain detectable in ART-experienced, virologically suppressed people with HIV after switching to either B/F/TAF or DTG/3TC, indicating that regimen optimization alone does not restore innate immune homeostasis. The enhanced expression of migratory and HIV-related receptors suggests ongoing reservoir maintenance and residual inflammation.

## 1 Introduction

The DEBATE study is a randomized clinical trial (RCT) designed to evaluate and compare immunological outcomes in people with HIV (PWH) who maintain viral suppression. The trial investigates the effects of switching antiretroviral therapy (ART) from a standard three-drug regimen (3DR) to either a two-drug regimen (2DR) consisting of dolutegravir/lamivudine (DTG/3TC) or to an alternative three-drug regimen composed of bictegravir/emtricitabine/tenofovir alafenamide (B/F/TAF) (1).

B/F/TAF is a guideline-recommended regimen for the treatment of HIV-1 infection in both naïve and ART-experienced PWH. DTG/3TC is the first dual ART to be included in all international guidelines as a recommended regimen for PWH, whether they are ART-naïve or experienced.

This 2DR has been evaluated in several trials in both ART-naïve patients (GEMINI-1 and GEMINI-2) and virologically suppressed patients (TANGO and SALSA). In these large randomised clinical trials, DTG/3TC was shown to be non-inferior to 3DRs in terms of virological outcomes, and this dual regimen is currently widely used in clinical practice (2–5).

In the DEBATE trial, over 12 months, no significant differences were observed between the two groups in absolute CD4^+^ and CD8^+^ T-cell counts, CD4/CD8 ratio, or plasma IL-6 levels, indicating overall immunological stability following regimen simplification. Nevertheless, more detailed immunophenotypic analyses revealed subtle differences. Participants switching to DTG/3TC displayed increased proportions of transitional memory and terminally differentiated CD4^+^ T cells at six months, with similar trends observed in CD8^+^ T-cell subsets. Additionally, a transient reduction in non-classical monocytes was noted in the B/F/TAF group at six months, which was no longer observed at twelve months.

Collectively, these findings suggest that switching to DTG/3TC maintains overall immune homeostasis comparable to B/F/TAF, while inducing modest shifts in specific T-cell differentiation and monocyte subsets. The clinical significance of these immunological changes warrants further investigation (1).

In recent years, most of the study have focused on the evaluation of T lymphocytes recovery and remodeling upon HIV treatment (6, 7), but very little attention has been given to the innate immune compartment. Monocytes play a pivotal role in orchestrating inflammatory responses, sensing viral components, and bridging innate and adaptive immunity, and they also contribute to the persistence of the viral reservoir (8). There is increasing evidence that monocyte activation persists in ART-treated PWH. This includes alterations in the distribution of monocyte subsets, increased expression of activation markers and interferon-stimulated genes, higher production of proinflammatory cytokines, and elevated levels of soluble inflammatory markers such as sCD14 and sCD163 (9). These abnormalities have been linked to microbial translocation, residual immune stimulation by viral antigens, and chronic exposure to inflammatory mediators (10). Persistent monocyte activation has been associated with a wide range of non-AIDS comorbidities, including cardiovascular disease, neurocognitive impairment, and metabolic disorders, highlighting its clinical significance even in individuals with fully suppressed viremia (11, 12).

To better characterize the remodeling of the innate immune compartment after long-term viral suppression, we conducted a sub-study of PWH enrolled in the DEBATE trial, in which monocyte phenotype was analyzed by high-dimensional flow cytometry at baseline and 36 months after the switch.

## 2 Materials and methods

### 2.1 Study design

Participants included in the present study were selected from the DEBATE trial cohort. Eligibility for inclusion in the current analysis was based on the availability of an adequate baseline cryopreserved peripheral blood mononuclear cells (PBMC) sample stored in the biobank from the original DEBATE study. Thus, only participants for whom a baseline sample (T0) was still available and suitable for the present experimental analyses were included (1). Paired samples collected at baseline (T0) and at the 36-month follow-up (T3) were analyzed.

The study (EudraCT number 2018-003458-26; ClinicalTrials.gov identifier NCT04054089) was approved by the local Ethics Committee (EC) (authorization number AOU 0010923/19, April 4, 2019) and by the Italian Medicines Agency (AIFA) (authorization number AIFA/SC/P/33830, March 25, 2019). The official start date of the DEBATE study, following approval by the General Director of the University Hospital (AOU), was May 14, 2019. The study ended on July 28, 2022, as notified to the EC and AIFA.

All participants provided written informed consent for sample collection and data analysis. Demographic and clinical characteristics of the participants are reported in **Table 1**.

**Table 1:** Demographic and clinical characteristics of PWH.

PWH (n)	11
Males (n, %)	11, 100
Females (n, %)	0, 0
age (y, mean ± st. dev)	46.36 ± 10.72
Time since HIV diagnosis, years, median (Q1, Q3)	15.19 (Q1, Q3: 8.78, 20.04)
Time since DEBATE switch, years, median (Q1, Q3)	2.96 (Q1, Q3: 2.73, 3.05)
Time since HIV RNA undetectability in the DEBATE, years, median (Q1, Q3)	2.96 (Q1, Q3: 2.73, 3.05)
Time since HIV RNA undetectability (overall), years, median (Q1, Q3)	10.03 (Q1, Q3: 8.33, 14.78)
Therapy switch	
B/F/TAF (n, %)	5, 45.45
DTG/3TC (n, %)	6, 54.55
Adverse events	
Fever (n, %)	2, 18.18
Memory decline (n, %)	1, 9.09
Bursitis (n, %)	1, 9.09
None (n, %)	7, 63.64

### 2.2 Blood collection and isolation of mononuclear cells

Up to 30 mL of blood was collected from PWH in Vacuette tubes containing ethylenediaminetetraacetic acid (EDTA) and processed immediately. PBMC were isolated by Ficoll-Hypaque density-gradient centrifugation (Lymphoprep, Sentinel Diagnostics) according to standard procedures. PBMC were stored in liquid nitrogen in fetal bovine serum (FBS) supplemented with 10% dimethyl sulfoxide (DMSO). Plasma was stored at −80 °C until use.

### 2.3 Multicolor flow cytometry detection of surface and intracellular antigens of monocytes

For *ex vivo* immunophenotyping experiments, cryopreserved PBMC were thawed in RPMI 1640 medium supplemented with 10% fetal bovine serum (FBS), 1% L-glutamine, 1% sodium pyruvate, 1% nonessential amino acids, antibiotics, 0.1 M 4-(2-hydroxyethyl)-1-piperazineethanesulfonic acid (HEPES), and 55 mM β-mercaptoethanol, referred to as R10, further supplemented with 20 μg/ml DNase I from bovine pancreas (Sigma-Aldrich), were washed with PBS and stained with viability marker Viakrome 808 (Beckman Coulter, Hieleah, FL) and FC-receptors blocker Human TrueStain FcxTM (Biolegend, catalog number: 426103) for 20 min at room temperature (RT). Next, cells were washed with FACS buffer (PBS with 2% of FBS) and stained with following surface mAbs for chemokines detection: CX3CR1-BB700, CCR2-BV510, CCR5-BV650, CXCR4-PE-CY5 in 1:40 of True Stain Monocytes Blocker (Biolegend, catalog number: 426103) and 1:1 FACS: Brillant Stain buffer (BD, catalog number: 566349), and incubated 30 min at 37 °C, 5% CO_2_. Then, cells were washed with FACS buffer and stained with following surface mAbs: CD3-PE/DAZZLE 594, CD66b- PE/DAZZLE 594, CD19- PE/DAZZLE 594, CD56- PE/DAZZLE 594, CD123- PE/DAZZLE 594, CD1c- PE/DAZZLE 594, HLA-DR-BUV661, CD14-BUV395, CD16-BV605, MerTK-BV421, Axl-APC, CD209-APC/FIRE750, CD38-BUV496, TREM-2-AF790, Tyro3-PE in 1:40 of True Stain Monocytes Blocker and 1:1 FACS: Brillant Stain buffer, and incubated 20 mins at RT. After the incubation, cells were washed with FACS buffer and fixed and permeabilized with eBioscience Intracellular fixation and permeabilization Buffer set (Thermofisher, catalog number: 88-8824-00) according to manufacturer’s instructions. Then, cells were stained with following mAbs recognizing intracellular antigens: p16-VioB515, pSTAT3-PE-CY7 and incubated 30 mins at RT.

Paired T0 and T3 samples from the same participant were thawed, stained, and acquired within the same experimental batch to minimize inter-assay and technical variability.

To maximize the number of channels available for monocyte phenotyping, six non-monocytic lineage markers (CD3; CD19; CD56; CD66b; CD123; and CD1c) were conjugated to PE-Dazzle to serve as a consolidated dump channel. Detailed mAbs information is reported in **Table 2**. Samples were acquired at Attune NxT Xenith spectral flow cytometer (Thermofisher), according to current guidelines (13).

**Table 2:** Monocyte flow cytometry panel configuration.

Target	Dye	Clone	Manufacturer	Catalogue number	Lot number	Titer (uL/100 uL)
Live/Dead Near IR 876		N/A	Thermofisher	L34980	/	1.25
CD66b	PE-DAZZLE	G10F5	Biolegend	C36628	B392324	2.5
CD3	PE-DAZZLE	UCHT1	Biolegend	A20189	B357812	2.5
CD19	PE-DAZZLE	HIB19	Biolegend	304045	B397366	2.5
CD56	PE-DAZZLE	HCD56	Biolegend	A20189	B380143	2.5
CD123	PE-DAZZLE	6H6	Biolegend	305122	B370590	2.5
CD1c	PE-DAZZLE	L161	Biolegend	300450	B345894	2.5
CD14	BUV395	63D3.RMAB	BD	302252	4143145	5
CD16	BV605	3G8	Biolegend	318348	B399752	1.25
HLA-DR	BUV661	G46-6	BD	306034	7249926	1.25
CX3CR1	BB700	2A9-1	BD	331532	4165949	5
p16	VioB515	REA973	Miltenyi Biotech	130-130-645	5240601010	5
MerTK	BV421	590H11G1E3	Biolegend	302040	B406914	5
Axl	APC	108724	Biotechne R&D	612980	ADDW0423041	5
Tyro3	PE	96201	Biotechne R&D	746135	LBF0623091	5
p-STAT3 (Ser727)	PE-Cy7	A16089B	Biolegend	698908	B395096	5
CD209	APC/FIRE750	9E9A8	Biolegend	367604	B355491	5
CCR5 (CD195)	BV605	2D7/CCR5	BD	FAB154A	4219035	2.5
CCR2 (CD192)	BV510	LS123.1D9	BD	FAB859P	4219039	2.5
CXCR4 (CD184)	PE-CY5	12G5	Biolegend	698908	B386017	2.5
CD38	BUV496	HIT2	BD	612946	1077726	0.6
TREM-2	AF790	B-3	SantaCruz (DBA)	sc-373828 AF790	E0124	1.25

The panel comprises 17 parameters, comprising 16 fluorescence detection channels and a viability dye, and 21 fluorochrome-conjugated antibodies to resolve 15 target antigens. Six lineage exclusion markers (CD3, CD19, CD56, CD66b, CD123, CD1c) were consolidated into a single PE-Dazzle Dump channel to ensure clean exclusion of non-monocytic populations without using additional detection channels.

### 2.4 Computational analysis of flow cytometry data

Compensated Flow Cytometry Standard (FCS) 3.0 files were imported into FlowJo software version v10.7.1 and analyzed by standard gating to remove doublets, aggregates and dead cells. For each sample, all live CD14+HLA-DR+ cells were exported and imported into R using the flowCore package v2.4.0, yielding a total of 4,801,385 CD14+HLA-DR+ cells across all samples and timepoints. Subsequent analyses were performed using CATALYST v1.17.3. All flow cytometry data were transformed in R using the hyperbolic arcsine function, “arcsinh(x/cofactor)”, with manually defined cofactors, where x represents the measured fluorescence intensity.

Clusters were subsequently identified based on the expression of additional markers known to characterize classical, intermediate, and non-classical monocytes. Classical monocytes were identified as the cluster displaying high expression of CCR2 and CXCR4, together with high CXCR5 expression and absent or very low CX3CR1 expression. Intermediate monocytes were characterized by reduced CCR2 and CXCR4 expression and increased CX3CR1 expression, whereas non-classical monocytes were identified by absent or very low CCR2 and CXCR4 expression and high CX3CR1 expression (14, 15).

To enable robust visualization and facilitate biological interpretation across treatment conditions and timepoints, we performed stratified downsampling of the full dataset. Specifically, we selected 50,000 cells per condition (B/F/TAF and DTG/3TC) and timepoint (T0 and T3), yielding a balanced subset of 200,000 cells for dimensionality reduction. This stratified approach ensures equal representation of each experimental group, thereby preventing visualization bias toward larger populations and enabling fair comparative analysis. Clustering and dimensional reduction were subsequently performed on this stratified subset using FlowSOM (version 2.4.0) and UMAP (version 0.2.8.0) algorithms, respectively, with a fixed random seed to ensure reproducibility.

### 2.5 Statistical analysis

Principal component analysis (PCA) was performed on the z-scored monocyte parameters (frequencies and MFI) obtained by manual gating. Multivariate differences were assessed by PERMANOVA (adonis2, vegan package) on Euclidean distances between z-scored parameter values. Because each patient was sampled at both timepoints, permutations were restricted within patients for the time effect, and whole patients were permuted as units for the between-regimen comparison. Homogeneity of multivariate dispersion was verified by PERMDISP (betadisper). Analyses were performed in R with a fixed random seed. Univariate differences in marker frequencies and MFI were assessed by two-way ANOVA for matched (paired) values. p values< 0.05 were considered statistically significant.

## 3 Results

Eleven virally suppressed PWH were included in the analyses: six switched to DTG/3TC and five to B/F/TAF. Their demographic and clinical characteristics are shown in **Table 1**. All participants remained clinically stable throughout the observation period, and no clinically relevant episodes of cytomegalovirus reactivation were detected during follow-up.

Principal component analysis of the first two components, accounting for 25.8% and 20.5% of the total variance, showed a separation between baseline (T0) and follow-up (T3) samples (**Figures 1A, C**). The loading plot identified frequency and mean fluorescence intensity (MFI) changes in CXCR4, CCR5, CX3CR1 and MerTK as the main contributors to this separation (**Figure 1B**).

**Figure 1:**
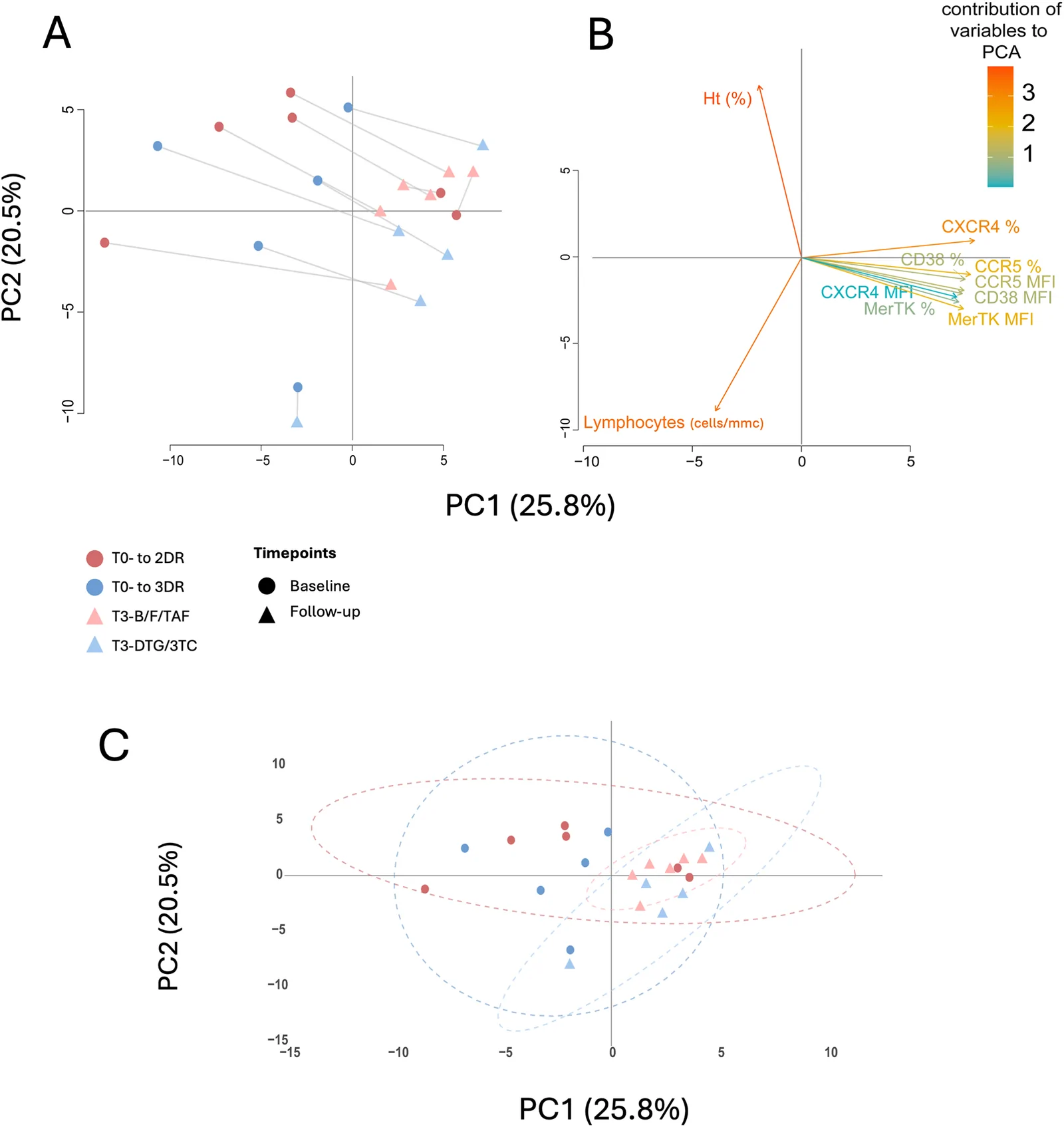
Principal component analysis (PCA) of patient samples at baseline (T0, circles) and 3 months post-therapy switch (T3, triangles). **(A)** PCA showing the distribution of patients initially receiving a 3-drugs regimen (blue) switching to B/F/TAF (light blue), and PWH initially receiving another 3-drugs regimen (red) switching to DTG/3TC (pink). The first two principal components account for 25.8% and 20.5% of the total variance (46.3% combined; all components are reported in **Table 3**). Dashed ellipses represent 95% confidence regions and are shown for visualization purposes only; differences between timepoints and therapy regimens were formally assessed by PERMANOVA on the full set of measured variables (**Table 4**). **(B)** Plot displaying the variables as vector, indicating the direction of each variable to overall distribution. The strength of each variable is represented by colors: orange color represents a strong contribution; light blue color represents a milder contribution. Length and direction of the arrows indicate the weight and correlation for each parameter. **(C)** PCA showing the distribution of patients initially receiving a 3-drugs regimen (blue) switching to B/F/TAF (light blue), and PWH initially receiving another 3-drugs regimen (red) switching to DTG/3TC (pink). Ht: hematocrit. PERMANOVA statistical test has been applied assessing significant differences between therapy regimens and timepoints across the multivariate profile of measured variables (**Table 4**).

Since the first two components accounted for only 46.3% of the total variance, and the first four and first ten for 68.3% and 91.4% respectively (**Table 3**), differences were tested by PERMANOVA on the full set of measured parameters rather than on component scores (**Table 4**). Monocyte phenotype differed significantly between T0 and T3 (R^2^ = 0.175, p = 0.0065, permutations restricted within patient), and PC1 shifted in the same direction in 10 of 11 patients (p = 0.011), indicating a consistent change at the individual level. The two regimens were indistinguishable at baseline (p = 0.78) and differed at T3 (R^2^ = 0.199, p = 0.023), but the magnitude of the within-patient change did not differ between them (p = 0.43); given the small group sizes, this comparison is considered exploratory.

**Table 3:** Percentage of variance and eigenvalue explained by each principal component.

Principal component (PC)	Eigenvalue	Variance (%)	Cumulative variance (%)
PC 1	12.38	25.79	25.79
PC 2	9.83	20.48	46.27
PC 3	5.68	11.83	58.1
PC 4	4.88	10.18	68.27
PC 5	2.66	5.54	73.81
PC 6	2.16	4.51	78.32
PC 7	2.06	4.29	82.61
PC 8	1.54	3.21	85.82
PC 9	1.43	2.97	88.8
PC 10	1.23	2.57	91.36
PC 11	0.99	2.06	93.42
PC 12	0.72	1.5	94.92
PC 13	0.5	1.04	95.96
PC 14	0.43	0.9	96.86
PC 15	0.41	0.85	97.72
PC 16	0.31	0.65	98.36
PC 17	0.29	0.6	98.97
PC 18	0.21	0.44	99.4
PC 19	0.15	0.31	99.71
PC 20	0.09	0.19	99.9
PC 21	0.05	0.1	100

**Table 4:** Permutational multivariate analysis of variance (PERMANOVA) assessing the effects of time point, treatment regimen, and their interaction on the multivariate data.

Comparison	Df	R^2^	F	Pr(>F)
Between-patient variation	10	0.559	2.094	NA
Time point (T0 vs T3)	1	0.175	6.549	0.0065
Time point, unconditioned	1	0.175	4.234	0.007
Regimen, overall	1	0.056	1.181	0.417
Regimen at T0	1	0.056	0.53	0.7846
Regimen at T3	1	0.199	2.232	0.023
Regimen x time point	1	0.095	0.941	0.4316
Between-patient variation (48 vars)	10	0.622	2.541	NA
Time point, all 48 variables	1	0.133	5.434	0.005

The analysis reports degrees of freedom (Df), coefficient of determination (R^2^), pseudo-F statistic (F), and permutation-based p-values (Pr(>F)). Between-patient variation was included to account for inter-individual variability. Comparisons were performed between T0 and T3, both overall and stratified by treatment regimen. The analysis was also repeated considering all 48 variables. NA, not applicable.

### 3.1 Monocyte phenotype remodeling and involvement in HIV-triggered immune escape, chronic activation and senescence

A 17-parameter flow cytometry panel was applied and total monocytes, identified as CD14^+^HLA-DR^+^ cells, were analyzed by an unsupervised method, *i.e.*, FlowSOM, to better depict their phenotype (**Figures 2A–C**). Clustering identified six populations spanning the entire monocyte compartment: one cluster of classical monocytes (C1, CCR2^++^CCR5^++^CD14^++^HLA-DR^++^CXCR4^++^), three clusters of intermediate monocytes (C2, C3 and C5, CCR2^+^CD14^+^CXCR4^+^) and two clusters of non-classical monocytes (C4 and C6, CCR2^-^CD14^dim^CX3CR1^++^CXCR4^-^).

**Figure 2:**
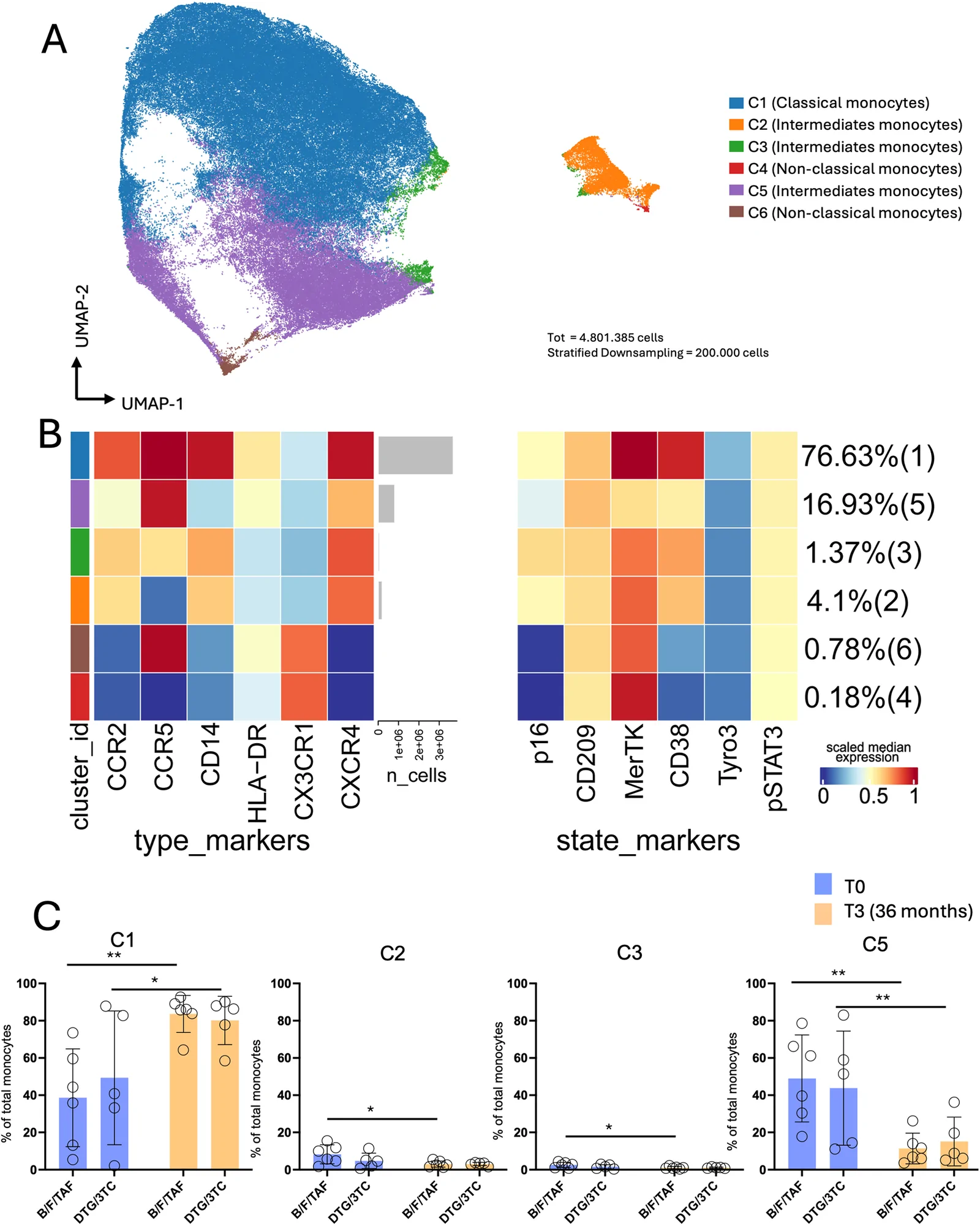
Monocyte landscape. **(A)** UMAP representing monocyte phenotype. **(B)** Heatmap representing monocyte phenotype. **(C)** Dot plots of different subpopulations in B/F/TAF- and DTG/3TC-treated PWH. Two-way Anova test for matched (paired) values was used to calculate statistical significance, p-value was indicated by * p< 0.05, ** p< 0.01, and *** p< 0.001.

Across all clusters, a generally activated phenotype was characterized by the expression of MerTK and CD209 (DC-SIGN). CD209 is a C-type lectin that can mediate HIV internalization and protect the virus from degradation (16, 17), a process also sustained by MerTK-mediated phagocytic activity (18), thus promoting HIV infection and immune escape.

Either B/F/TAF- and DTG/3TC- treated PWH show an increase in the classical subset expressing CD209, MerTK and senescent and activation markers like p16 and CD38 (C1) after 36 months. On the other hand, the same PWH show a decrease in the intermediate subset expressing CD209, MerTK and pSTAT3 (C5). In particular, PWH treated with B/F/TAF show a significant decrease in two of the intermediate clusters (C2 and C3) characterized by the expression of high levels of MerTK as well as CD209, p16 and CD38. This suggests a chronically activated, CD38-driven phenotype that may contribute to the establishment of a senescent phenotype characterized by p16 upregulation (19) and, potentially, to the maintenance of the viral reservoir.

### 3.2 CX3CR1, TAM receptors and CD38 role in monocytes activation and immune senescence

Manual gating (**Figures 3A, B**) revealed persistent monocyte activation, with upregulation of CX3CR1 frequency and median fluorescence intensity (MFI) regardless of the ART regimen, as well as of CD38 and of the MFI of MerTK and Tyro3 (**Figures 4A, B**, **5**). These effects were particularly pronounced after the switch to B/F/TAF. Both CX3CR1 and TAM receptors (MerTK and Tyro3) are closely associated with monocyte migration and the resolution of inflammation, as well as with senescence (20). Notably, CX3CR1 is downregulated by the HIV-1 Tat protein in microglia (21), a process that attenuates local inflammatory responses. This observation suggests that circulating monocytes may use CX3CR1-mediated pathways to promote systemic inflammation (22), potentially counterbalancing localized anti-inflammatory environments. Previous studies have linked elevated CD38 expression on CD8^+^ and CD4^+^ T cells to poor clinical outcome and ART failure, along with mitochondrial dysfunction (23). Similarly, persistent CD38 upregulation in monocytes, in terms of both MFI and cell frequency, may contribute to monocyte-driven tissue damage and immune senescence, thereby perpetuating chronic inflammation.

**Figure 3:**
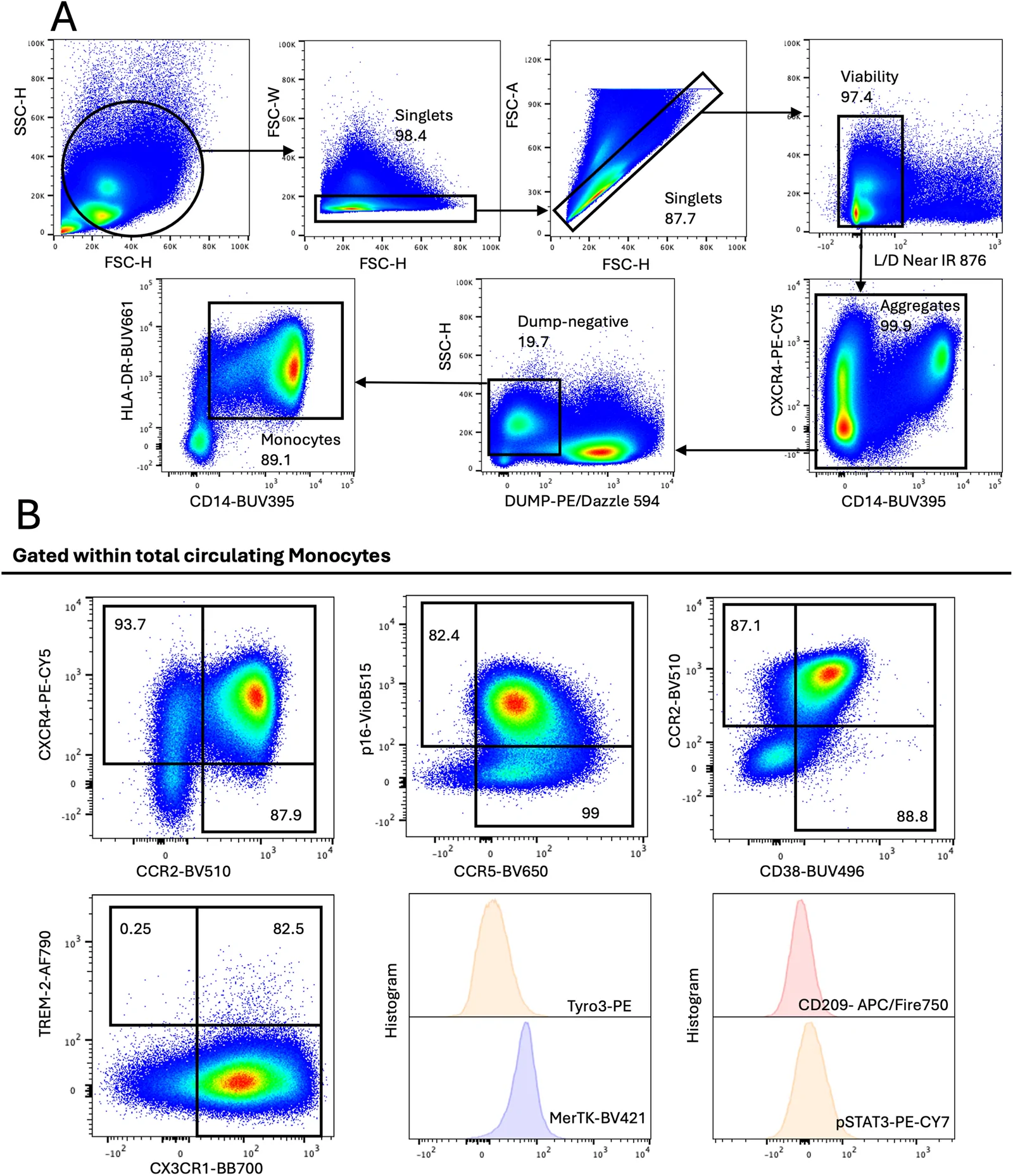
Gating strategy used to characterize monocyte phenotype. **(A)** Forward and side scatter (FSC and SSC) gating is used to identify cells of interest based on the relative size and complexity of the cells, while removing debris and other events that are not of interest. Further gating is done in an FSC-H, FSC-A and FSC-Width dot plot to eliminate doublets. Live/Dead Near IR876-population is used to select viable cells. On a bivariate plot of DUMP vs SSC-H DUMP- (CD66b/CD3/CD19/CD56/CD123/CD1c-) cells are selected. CD14^+^ HLA-DR^+^ monocytes are selected. **(B)** Expression levels of chemokines receptors (CCR5, CX3CR1, CXCR4, CCR2), TAM receptors (Axl, Mer, Tyro3), pSTAT3, p16, CD209, CD38 and TREM-2 are evaluated within total circulating monocytes.

**Figure 4:**
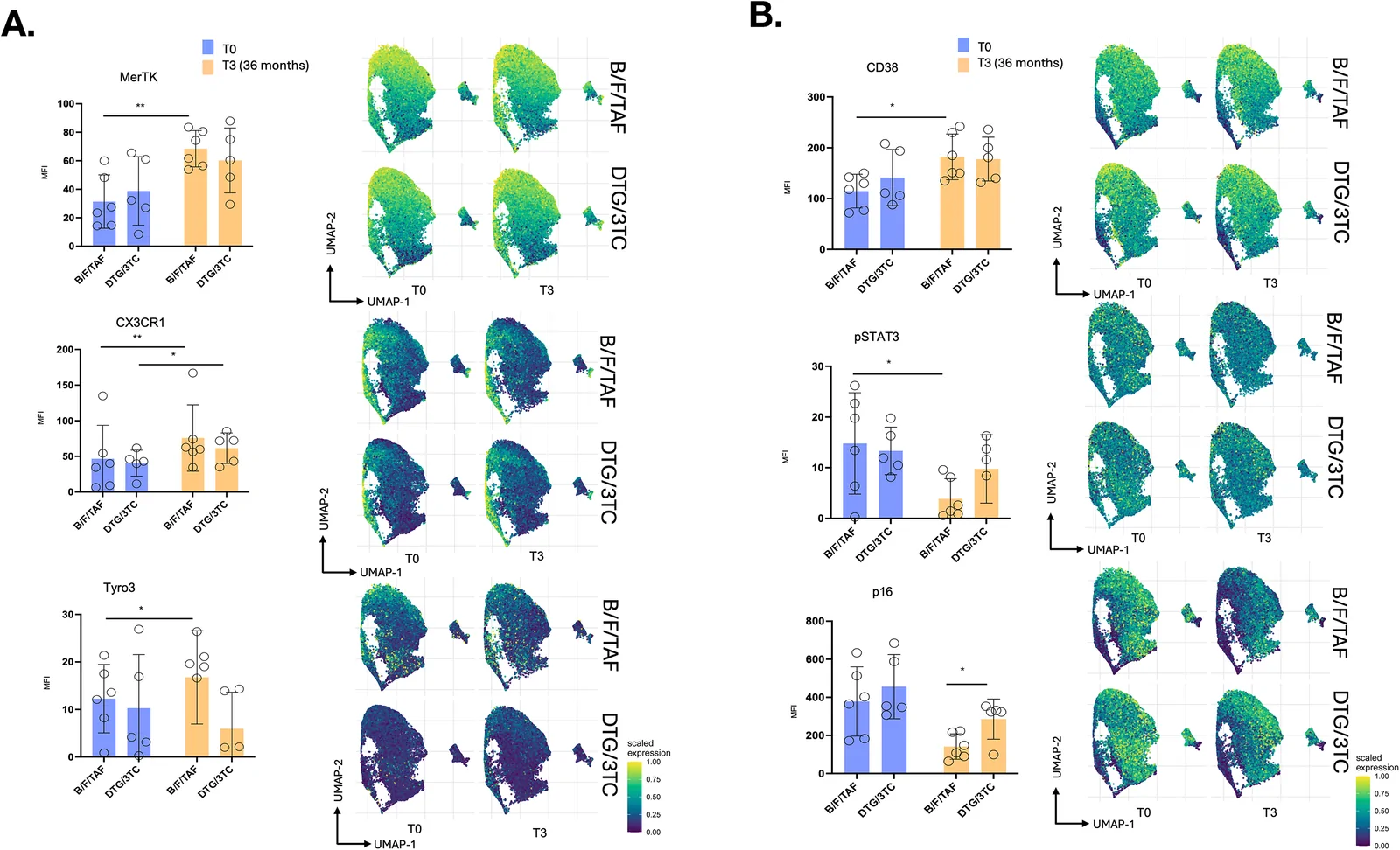
**(A)** Median fluorescence intensity (MFI) and UMAP of MerTK, CX3CR1 and Tyro3 in monocytes of PWH switching to B/F/TAF or DTG/3TC obtained by manual gating. Two-way Anova test for matched (paired) values was used to calculate statistical significance, p-value was indicated by * p< 0.05, ** p< 0.01, and *** p< 0.001. **(B)** Median fluorescence intensity (MFI) and UMAP of CD38, pSTAT3 and p16 in monocytes of PWH switching to B/F/TAF or DTG/3TC obtained by manual gating. Two-way Anova test for matched (paired) values was used to calculate statistical significance, p-value was indicated by * p<0.05, ** p< 0.01.

**Figure 5:**
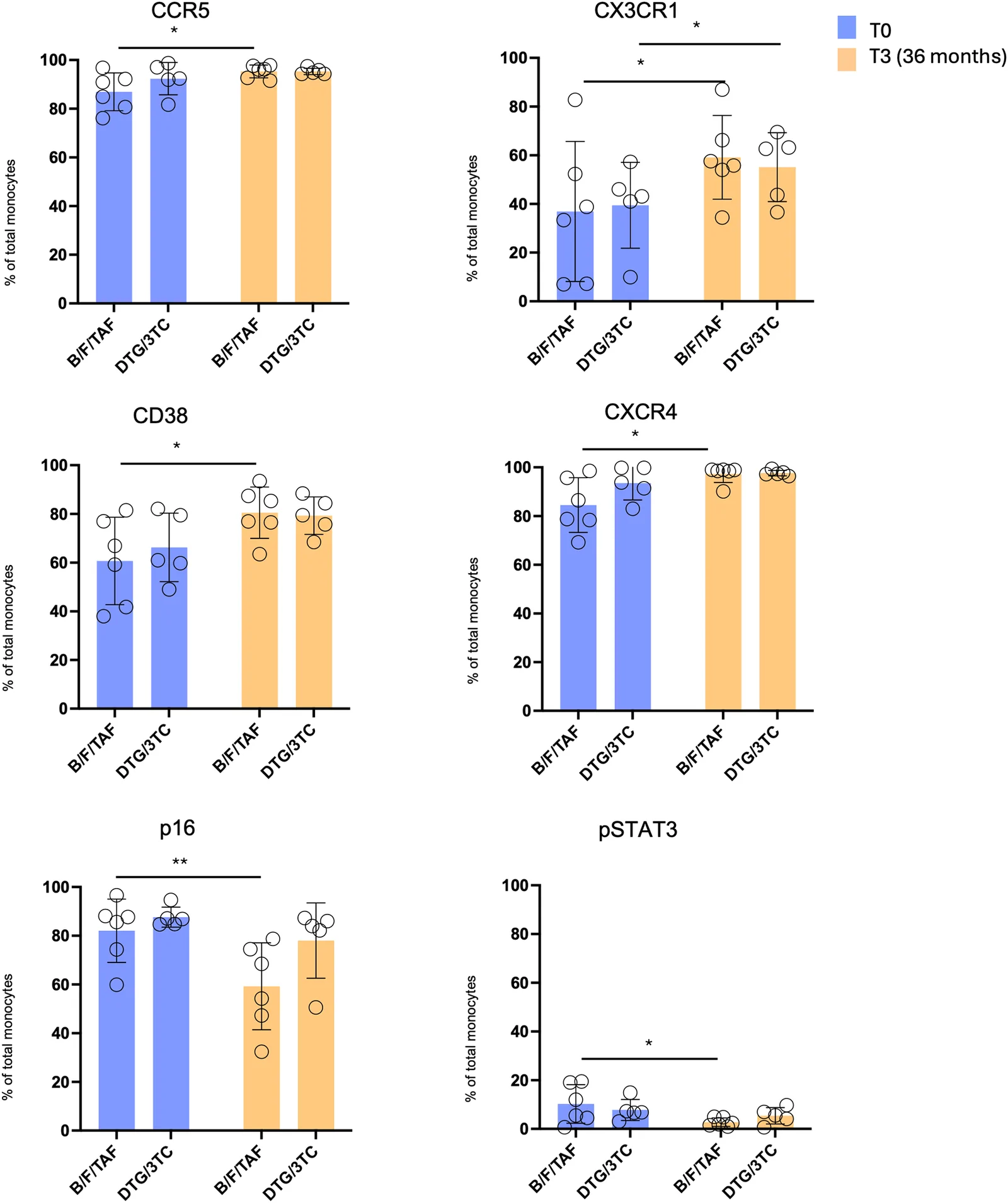
Frequencies of monocytes expressing CX3CR1, MerTK, Tyro3, CD38, pSTAT3, p16, CCR5 and CXCR4 in PWH switching to B/F/TAF or DTG/3TC, obtained by manual gating. Statistical significance was assessed by two-way ANOVA for matched (paired) values: *p < 0.05, **p < 0.01.

### 3.3 CCR5 and CXCR4 involvement in chronic inflammation and potentially in viral reservoir maintenance

Increased frequencies of CCR5^+^ and CXCR4^+^ monocytes were found at follow-up, especially after the switch to B/F/TAF (**Figure 5**). Besides serving as HIV co-receptors for viral entry, these receptors, once engaged, activate monocytes, enhancing the production of pro-inflammatory cytokines such as TNF, IL-1β and IL-6, as well as tissue migration. Consequently, these monocyte populations may contribute to both systemic inflammation (24) and the maintenance of the viral reservoir. In addition, CCR5 upregulation has been associated with higher D-dimer levels, suggesting a potential role of monocytes in non-AIDS-related complications, such as coagulopathies, in chronic HIV infection (25, 26). In contrast, decreased frequency and MFI of phosphorylated STAT3 (pSTAT3) (**Figures 4B**, **5**), reflecting reduced JAK-STAT signaling, as well as decreased p16 (**Figure 4B**), were detected. Reduced STAT3 phosphorylation has been associated with an impaired antiviral response, as observed in SARS coronavirus-infected cells (27), potentially exacerbating viral pathogenesis. Moreover, engagement of the pSTAT3 pathway is linked to acute immune activation (28), especially in the tumor microenvironment (29); thus, pSTAT3 downregulation in chronically stimulated monocytes might further support the long-term maintenance of the HIV reservoir.

## 4 Discussion

Our study shows that ART-experienced PWH undergo a profound remodeling of the innate immune compartment, particularly of monocytes. HIV-related immune senescence in ART-treated people has been widely studied, with most attention focused on CD8^+^ and CD4^+^ T cells (30–32). However, despite the known role of monocytes and macrophages in maintaining the latent HIV reservoir (33, 34) and chronic inflammation, which also drives HIV-related complications (35, 36), little attention has been paid to their phenotypic adaptation.

Monocyte immune senescence appears to be driven by migration markers, e.g., CXCR4, CCR5 and CX3CR1, and inflammatory markers, e.g., CD38, which may also contribute to HIV reservoir retention, especially in PWH treated with B/F/TAF, suggesting a potential benefit of simplifying long-term therapy to a 2DR such as DTG/3TC. However, chronic inflammation in DTG/3TC-treated people might be driven by HIV Nef-induced activation of the STAT3 pathway (37), as well as by IL-6 release following STAT3 activation (38). Although our data do not establish a direct association between monocyte pSTAT3 expression and HIV reservoir size or persistence, increased pSTAT3 may reflect sustained cytokine-driven JAK/STAT signaling and the chronic immune dysregulation that persists during treated HIV infection. Given the established role of this pathway in regulating immune-cell activation, survival, and homeostasis, persistent STAT3 activation may contribute to the cellular and inflammatory environment associated with HIV persistence (39–41). Whether increased pSTAT3 in monocytes directly or indirectly influences HIV reservoir maintenance remains to be determined and warrants further investigation.

The increased expression of MerTK and Tyro3 observed in monocytes may reflect their adaptation to a persistent inflammatory environment. Although TAM receptors are generally recognized for their immunoregulatory functions, increased MerTK expression has been reported in circulating myeloid cells in chronic inflammatory diseases. In patients with systemic lupus erythematosus, for example, MerTK expression was increased in circulating monocytes, particularly within the CD14^++^CD16^+^ subset, and correlated with systemic type-I interferon activity, suggesting that MerTK expression may be induced or maintained in response to inflammatory cues (42). In addition, studies in human monocytes have demonstrated that MerTK/Tyro3 signaling can modulate, and under certain conditions enhance, inflammatory cytokine production in response to LPS (43). These observations suggest that increased MerTK and Tyro3 expression may represent the engagement of compensatory regulatory pathways in chronically activated monocytes, potentially reflecting an attempt to restrain ongoing inflammation and promote tissue homeostasis while inflammatory signaling remains active. Thus, the simultaneous presence of inflammatory features and increased MerTK/Tyro3 expression may indicate a persistent, dysregulated monocyte state characterized by the coexistence of inflammatory activation and counter-regulatory mechanisms.

Taken together, these data indicate persistent monocyte activation after switching to either B/F/TAF or DTG/3TC, with more pronounced effects following the switch to B/F/TAF. This activation is characterized by a phenotype compatible with enhanced migratory capacity and, potentially, with ongoing maintenance of the viral reservoir. Switching from a 3DR to either another 3DR or a 2DR does not appear to normalize innate immune activation, underscoring the need for adjunctive strategies to mitigate residual inflammation in PWH.

These findings might have important clinical implications, as they show that virologically suppressed, ART-experienced PWH continue to exhibit persistent monocyte activation and immune senescence despite regimen optimization. The observed upregulation of migration- and activation-related receptors, including the HIV co-receptors CCR5 and CXCR4 and the chemokine receptor CX3CR1, together with TAM receptors, suggests sustained trafficking of activated monocytes to tissues and ongoing support of viral reservoir persistence, which may contribute to chronic inflammation and immune dysfunction. Such processes may be particularly relevant to the vascular and neurological compartments, where monocyte recruitment and activation have been implicated in atherosclerosis and HIV-associated neurocognitive impairment. Persistent monocyte alterations despite effective viral suppression may represent a pivotal biological link to residual non-AIDS morbidity in long-term treated HIV infection. Clinical observations consistently connect peripheral monocyte activation to organ-specific pathology; specifically, elevated monocyte activation markers correlate with cognitive impairment in virologically suppressed PWH (44), while monocyte-associated inflammatory profiles predict subclinical atherosclerosis, vascular remodeling, and systemic comorbidities (45, 46). Notably, although long-term ART restores monocyte subset proportions, it fails to resolve underlying cellular dysfunction, chronic low-grade inflammation, and substantial inter-individual phenotypic heterogeneity (47, 48). This discrepancy indicates that numerical recovery does not equate to functional normalization. In this context, sustained monocyte hyper-reactivity to secondary Toll-like receptor (TLR) stimulation, rather than a typical resolving immune response, is a hallmark of maladaptive trained immunity, linking non-resolving cellular activation to an increased risk of cardiovascular disease (49, 50). During our 36-month follow-up, none of the participants developed cardiovascular disorders, and only one displayed cognitive decline. However, this clinical stability and the lack of overt clinical events within this timeframe do not preclude the presence of underlying subclinical vascular or neuroinflammatory processes. Consequently, the persistence of an altered, subclinically primed monocyte phenotype under sustained ART may act as a driver of chronic tissue injury and non-communicable disease in aging PWH (51).

Our study has some limitations. In particular, the relatively small sample size did not allow an adequately powered separate analysis of the two therapeutic regimens. The comparison between regimens should therefore be regarded as exploratory: with five and six patients per group, the study was not powered to detect a regimen-specific effect, and the difference observed at T3 was not accompanied by a significant difference in the magnitude of within-patient change. Expanding the cohort in future studies would facilitate a clearer interpretation of these findings from a translational perspective. Furthermore, all enrolled participants were male. Therefore, the findings cannot be generalized to women, and potential sex-related differences in innate immune phenotypes cannot be excluded. Given the well-documented influence of biological sex on immune responses, further studies including both male and female participants are warranted to assess possible sex-specific immunological differences.

In addition, supervised monocyte phenotypic analysis was performed on total monocytes without stratification into classical, intermediate, and non-classical subsets. This methodological choice was dictated using cryopreserved PBMC. Cryopreservation is known to alter the surface expression of certain markers, including CD16 (52–54), which is critical for the discrimination of monocyte subsets. In frozen and subsequently thawed samples, CD16 expression is frequently downregulated or variable, thereby impairing the reliable identification and classification of monocyte subpopulations. Consequently, to avoid potential misclassification and ensure data consistency, analyses were conducted on the total monocyte population rather than on CD16-defined subsets. Monocyte clusters were subsequently identified by unsupervised analysis based on the differential expression of lineage-defining markers corresponding to classical, intermediate, and non-classical subsets. Classical monocytes were defined by high expression of CCR2, CXCR4, and CCR5, accompanied by minimal or absent CX3CR1 expression. Intermediate monocytes exhibited reduced levels of CCR2 and CXCR4 alongside elevated CX3CR1 expression. Conversely, non-classical monocytes were characterized by high CX3CR1 expression with little or no detectable CCR2 or CXCR4 (14, 15). Finally, our study design prioritized phenotypic profiling over functional evaluation, and the HIV reservoir was not measured; thus, although distinct phenotypic shifts in monocytes were observed, their direct physiological consequences remain to be fully elucidated.

The inability of both three-drug and two-drug regimens to normalize innate immune activation underscores the limitations of ART alone in fully restoring immune homeostasis and highlights the need for adjunctive therapeutic strategies targeting innate immune pathways to reduce inflammation, mitigate immune aging, and improve long-term clinical outcomes in PWH. In conclusion, these findings challenge the assumption that modern two- or three-drug regimens with a high barrier to resistance confer immunological equivalence beyond virological suppression, and they underscore the need to study adjunctive immunomodulatory strategies, such as anti-inflammatory agents, checkpoint inhibitors, or targeted senolytic approaches, to complement ART in addressing the residual immune dysfunction that persists despite optimal virological control.

## Data Availability

The raw data supporting the conclusions of this article will be made available by the authors, without undue reservation.
